# Visual Classification of Tau-PET Detects 4 Subtypes With Different Long-Term Outcomes

**DOI:** 10.1212/WNL.0000000000213950

**Published:** 2025-09-12

**Authors:** Cecilia Boccalini, Gregory Mathoux, Ines Hristovska, Federica Ribaldi, Debora Elisa Peretti, Annachiara Arnone, Max Scheffler, Giovanni Battista Frisoni, Oskar Hansson, Jacob W. Vogel, Valentina Garibotto

**Affiliations:** 1 Laboratory of Neuroimaging and Innovative Molecular Tracers (NIMTlab), Geneva University Neurocenter and Faculty of Medicine, University of Geneva, Switzerland;; 2 Division of Nuclear Medicine and Molecular Imaging, Geneva University Hospitals, Switzerland;; 3 Clinical Memory Research Unit, Department of Clinical Sciences Malmö, Faculty of Medicine, Lund University, Sweden;; 4 Geneva Memory Center, Department of Rehabilitation and Geriatrics, Geneva, University Hospitals, Switzerland;; 5 Laboratory of Neuroimaging of Aging (LANVIE), University of Geneva, Switzerland;; 6 Division of Radiology, Geneva University Hospitals, Switzerland;; 7 Department of Clinical Sciences Malmö, Faculty of Medicine, SciLifeLab, Lund University, Sweden; and; 8 CIBM Center for Biomedical Imaging, Geneva, Switzerland.

## Abstract

**Background and Objectives:**

Tau accumulation pattern shows substantial variability in Alzheimer disease (AD), and 4 distinct spatiotemporal trajectories were distinguished using a data-driven approach called the Subtype and Stage Inference (SuStaIn). A visual method to validate and identify these subtypes is a requirement for their clinical translation. Our study aimed to provide a standardized topographic method for identifying tau patterns visually using tau-PET in a clinical setting.

**Methods:**

Participants in this prospective study were included from the memory clinic of Geneva University Hospital. Inclusion criteria required participants to have undergone at least 1 ^18^F-Flortaucipir tau-PET scan and a Mini-Mental State Examination (MMSE) within a 1-year time frame. All scans were classified into different tau subtypes (limbic [S1], medial temporal lobe-sparing [S2], posterior [S3], and lateral temporal [S4]) using both visual rating and SuStain algorithm. A subgroup underwent amyloid-PET and clinical follow-up. Cohen's κ tested the agreement between raters and between visual and automated subtypes. Chi-squared and Kruskal-Wallis tests assessed differences in clinical and biomarker features between subtypes, whereas differences in cognitive trajectories were tested using linear mixed-effects models, controlling for age, sex, and clinical and tau stages.

**Results:**

A total of 245 tau-PET scans of individuals ranging from cognitively unimpaired to mild dementia (mean age: 68.25 years, 52% women) were included and classified into different tau pattern subtypes. A substantial agreement between raters was found in visually interpreting tau subtypes (κ > 0.65, *p* < 0.001) and a fair agreement between visual and automated subtypes (κ = 0.39, *p* < 0.001), with the automated approach more likely to classify a scan as tau negative and lower agreement between methods in more severe cases and AD clinical variants. Regarding the visual classification, individuals with S2 subtype were younger than S1 and S3, had lower MMSE and verbal fluency scores than S4 and S1, showed higher global tau burden than other subtypes, and a steeper cognitive decline.

**Discussion:**

Visual classification reliably identified 4 tau patterns that differ in global tau load, clinical features, and long-term outcomes, suggesting its clinical usefulness for the detection of higher-risk AD variants. A clinically implementable classification of subtypes with faster decline is paramount for personalized diagnosis, accurate prognosis, and treatment.

## Introduction

Alzheimer disease (AD) is a progressive neurodegenerative disease whose neuropathologic hallmarks are β-amyloid plaques and neurofibrillary tau tangles.^1^ Both neuropathologic features can be detected in vivo using PET. Tau-PET holds the potential to become an important diagnostic and prognostic tool in clinic setting given its strong association with clinical performance and cognitive decline. Tau-PET levels are strongly associated with cognitive changes in the preclinical and prodromal stages of AD,^2,3^ outperforming amyloid-PET, fluorodeoxyglucose (FDG)-PET, and structural MRI in direct comparisons.^3-5^ Among tau tracers, ^18^F-flortaucipir is the most widely used and the only US Food and Drug Administration (FDA)–approved test establishing biomarker evidence of AD in living people. Tau-PET looks also promising to optimize upcoming tau-targeting clinical trials, by assessing target engagement, selecting participants, and monitoring the treatment efficacy.^6^

In patients with AD, the group-level spatial and temporal distribution of tau-PET fits the characteristic pattern of tau propagation defined according to the neuropathologic hierarchic stages.^7,8^ However, not all individual tau patterns fit Braak staging system, and substantial variability in spreading patterns within AD population has been now recognized. Among AD atypical cases that do not fit Braak scheme, hippocampal sparing, and limbic-predominant AD subtypes have been pathologically defined accounting for about 25% of cases.^9-11^ Moreover, the clinical variants of AD, such as posterior cortical atrophy (PCA) and logopenic variant primary progressive aphasia (lvPPA), represent other examples of atypical tau patterns mirroring the clinical and neuroanatomical AD variability.^12^ More recently, 4 spatiotemporal tau patterns have been distinguished using a data-driven model called the Subtype and Stage Inference (SuStaIn) algorithm^13^: limbic (S1), medial temporal lobe (MTL)-sparing (S2), posterior (S3), and lateral temporal (S4) subtypes.^14^ These subtypes exhibit different clinical profiles and longitudinal outcomes, with the subtype expressing both cortical and MTL tau with marked lateralization (S4) exhibiting the most aggressive phenotype.

This subtype framework seems promising for selection in clinical trials, possibly allowing for more individualized clinical care and eventually treatments. However, there is an unmet need to translate these tau accumulation subtypes into a clinical setting. Although most available research data rely on semi-quantitative measures, visual interpretation methods are needed for clinical translation as indicated also by FDA instructions for tau-PET requiring visual reads.^15^ To the aim of translating tau subtypes to a clinical setting, our study addressed the validation of these subtypes and provides a visual method to clinically identify them. Specifically, our study aims to (1) present an algorithm for topographical visual assessment of tau accumulation subtypes and provide their evidence in a clinical setting, (2) evaluate the agreement between the subtype classifications obtained with the topographic visual method and the original SuStaIn method, and (3) assess differences in clinical, biomarker, and cognitive trajectories between the different subtypes.

## Methods

### Participants

We collected all ^18^F-Flortaucipir-PET scans of people referring to Geneva Memory Center and performed at Geneva University Hospitals (Switzerland) between 2016 and 2024. As inclusion criteria, participants needed to have completed a tau-PET scan and at least 1 Mini-Mental State Examination (MMSE) within a 1-year period. Each participant underwent the standard memory clinic workup, which includes assessments of clinical and neurologic status, MMSE, and MRI scans.^16^ Participants range from normal cognitive function (CU) to mild cognitive impairment (MCI) and dementia (DEM). CU participants were recruited from volunteers or patients referred to the memory clinic who exhibited normal cognition on all cognitive tests. Cognitively impaired participants were diagnosed with MCI or DEM based on standard clinical diagnostic criteria.^17,18^

A subset of participants (N = 215) also underwent amyloid-PET scans within 1 year of tau-PET assessment. APOE genotyping has been performed for a subgroup of 170 participants.

A more detailed neuropsychological battery was available for a subset of participants, with a higher percentage of test availability in CU and MCI, than DEM. Cognitive testing included the Free and Cued Selective Reminding Test 16-item version with immediate recall (N = 170), delayed free and delayed total recall (N = 143) to assess episodic memory; Trail-Making Test part A (N = 159) and part B (N = 140) to assess executive function; Semantic (N = 135) and Phonemic Fluency (N = 131) to assess executive abilities-working memory-language; and Digit Span (from Wechsler Adult Intelligence Scale-IV) (N = 161) to assess working memory.

### Standard Protocol Approvals, Registrations, and Patient Consents

The local Ethics Committee approved the imaging studies (No. 2016-01346), which have been conducted according to the principles of the Declaration of Helsinki and the International Conference on Harmonization of Good Clinical Practice. Each participant or their relatives provided voluntary written informed consent to participate in the studies.

### Imaging Acquisition and Preprocessing

All the images were acquired at Geneva University Hospitals, and imaging acquisition details are available in eMethods.

Amyloid-PET data preprocessing procedures and Centiloid calculation have been described previously.^3^ A threshold of 19 centiloid value was applied to differentiate between amyloid-positive and -amyloid-negative participants.^19^ Tau-PET preprocessing was consistent with previous works applying the SuStaIn^14^: Each participant's mean PET image underwent rigid coregistration to its respective native T1-weighted MRI image and images were intensity-normalized using inferior cerebellar gray matter as reference region, resulting in standardized uptake value ratio (SUVR) images. FreeSurfer parcellations were used to extract mean SUVR within different regions of interest (ROIs) for each participant in the native space. A global SUVR was calculated from the entorhinal cortex, lateral occipital cortex, inferior temporal cortex, and amygdala,^20^ constituting the meta-ROI.

### SuStaIn

The SuStaIn algorithm has been described in detail in previous publications^13,14^ and described in eMethods.

For our study, we initially normalized our dataset using the data from Vogel et al.^14^ First, the choroid plexus SUVR was regressed out of the tau-PET data. This dataset was then integrated with the “multi-tau” data used in the study by Vogel et al.^14^ Subsequently, the data were transformed into “tau Z-scores” using the method outlined in the same study. Two component Gaussian mixture models were used to define “normal” and “abnormal” tau distribution for each ROI. Z-scoring of each ROI was then performed using the mean and SD of the participants classified under “normal” distribution. Ultimately, the SuStaIn model, as used in the study by Vogel et al.,^14^ was applied to our dataset without undergoing any modifications. For the identification and exclusion of potential false positives (i.e., negative scans incorrectly classified as having tau pathology), the same methodology as^14^ was used but in an automated-only fashion (the original methodology used a combination of automated and visual methods). Gaussian mixture models were fitted to both the left and right entorhinal and precuneus regions, and the model posteriors were used to define the probability that an ROI is part of an “abnormal distribution.” Participants who were subtyped but not exhibiting at least 90% likelihood of being tau-positive in at least 1 of these 4 ROIs were classified as “false positive” and excluded.

### Tau-PET Visual Interpretation Method

All tau-PET scans were independently assessed by 2 nuclear medicine physicians blind to the clinical data and in a random order. When readings differed, a third expert rater helped to get a consensus reading, which was then used for statistical analysis.

The midpoint of the scans' color scale was set to the predominant color in the inferior cerebellar cortex, in accordance with published recommendations.^15^

Braak stages were visually defined^21^ as follows: Participants were classified as Braak I–III positive when the ^18^F-Flortaucipir signal showed mild to moderate increase in the MTL and fusiform gyrus; as Braak IV positive when it extended to the lateral temporal cortex; Braak V positive when it extended to the parietal or frontal cortex; and finally, as Braak VI positive when it extended to the motor and primary visual cortex. Scans with cortical uptake above background in other regions not considered by Braak were classified as non-AD and excluded from further analyses. To dichotomize the scans based on visual assessment, stages 0 and I–III were defined as T-, and stages IV–VI were defined as T+.

All scans were then visually assessed following a predefined regional cortical binding system (0 = no binding; 1 = mild binding; 2 = intense binding) for 6 brain regions (MTL, lateral temporal, frontal, parietal, occipital, precuneus; bilaterally). The binding system partially followed the FDA-approved method developed by Avid Radiopharmaceuticals identifying whether there is or not contiguous radiotracer uptake greater than 1.65 times the cerebellar uptake.^15^ Mild binding (uptake intensity grade of 1) was defined where there was contiguous radiotracer uptake ∼1.65 times the cerebellar uptake and then intensity binding (intensity grade of 2) when it was greater than ∼2 times. Color-wise, after setting the predominant color of the inferior cerebellar cortex as the midpoint (green), the mild uptake (grade 1) corresponds to the yellow to orange color transition, while the intense uptake (grade 2) to the red to pink one (eFigure 1).

**Figure 1:**
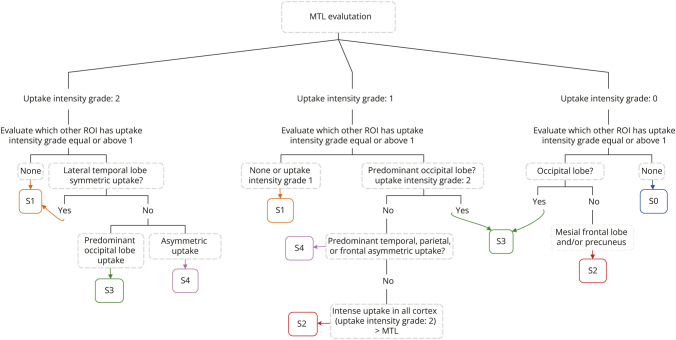
Visual Interpretation Method Diagram depicts the topographical visual interpretation method used to classify tau-PET scans into SuStaIn subtypes. S1 represents the limbic subtype usually fitting the Braak staging system, S2 represents the MTL-sparing subtype, S3 the posterior subtype, and S4 the lateral temporal. According to the 3-level scale for intensity biding rating, 0 is no binding, 1 means mild binding, and 2 intense binding. This binding intensity rating has been applied to different regions to consider the severity of tau load allowing us to identify regional predominance. MTL = medial temporal lobe; ROI = region of interest.

All scans were classified into SuStaIn subtypes: negative (S0), limbic (S1), MTL-sparing (S2), posterior (S3), and lateral temporal (S4) following the scheme detailed in Figure 1. The selected 6 regions represent the 10 spatial features of SuStaIn model, plus the precuneus representing an important AD-related region and a very early region of tau accumulation in MTL-sparing subtype.^14^ The 3-levels intensity scale simplifies the severity of tau load allowing to identify predominance of tau in certain regions over others. In this way, we aim to visually classify different subtypes at every stage. Individual examples of each subtype are shown in eFigure 2.

### Statistical Analyses

The proportion of individuals classified into each subtype according to the visual and the automated approaches was quantified. Cohen's κ was used as a measure of inter-rater agreement in the assignment of subtypes by raters and by approaches. The inter-rater agreement has been calculated also for each individual region. General linear models were performed to assess the correlation between algorithm-based stages and global tau SUVR.

Baseline demographics, clinical, cognitive, and biomarkers differences among subtypes were assessed using Kruskal-Wallis rank-sum test for continuous variables and a proportion test for categorical variables.

To obtain tau accumulation patterns, we performed voxel-wise *t* tests comparing tau SUVR images of each visual (and automated, separately) subtype against S0 (visual or automated) tau SUVR images, controlling for age and sex. The statistical threshold was set at *p* = 0.05, family-wise error-corrected at the cluster level.

We quantified the proportion of participants classified differently by the visual and automated approaches (discordant cases), and we compared discordant cases against the concordant cases (same classifications with the 2 approaches) in clinical and biomarker data.

To assess the prognostic value of subtypes obtained from different approaches, linear mixed-effects models were applied, including random intercepts and slopes, with longitudinal MMSE scores as the dependent variable, and either visual or automated subtypes as predictors. Age, sex, clinical stages, and visual Braak stages were included as covariates in the analysis to consider their possible influence on cognitive outcomes over time. Additional analyses including education and SuStaIn stages instead of Braak stages and excluding both were run.

All statistical analyses were performed using R, version 4.0.2.^22^ A *p* value of 0.05 was considered the significance threshold. Voxel-wise analyses were run in SPM12, and results were visualized using BrainNet Viewer software.^23^

### Data Availability

Anonymized data used in this study are available on reasonable request from the corresponding author (V. Garibotto).

## Results

### Sample Characteristics

Out of the total sample of 245 participants (Table 1), 72 were CU, 126 were diagnosed as MCI,^17^ and 47 as DEM.^24^ 52% were women, and the average age ± SD was 68.25 ± 4.54 years. Among amyloid-positive patients with MCI or DEM (N = 105), 87 (82%) presented clinically with a typical AD profile, while 16 (15%) presented clinically as atypical AD variants: lvPPA (N = 10), behavioral (N = 5), and PCA (N = 1). The remaining 2 individuals with MCI had mixed AD/DLB.

**Table 1:** Clinical Features of Visual Subtypes

	S0: Negative (N = 120)	S1: Limbic subtype (N = 64)	S2: MTL-sparing subtype (N = 23)	S3: Posterior subtype (N = 16)	S4: Lateral temporal subtype (N = 18)	*p* Values
Demographic features						
Age, y	69.4 (8.80)	73.5 (7.89)	64.4 (11.1)	75.7 (5.03)	72.6 (8.68)	<0.001^1,3,5,8^
Sex (women/men)	62/58	30/34	15/8	9/7	10/8	0.647
Education, y	13.9 (4.44)	13.9 (3.81)	12.2 (4.13)	13.9 (4.06)	12.8 (4.25)	0.396
Diagnostic stages (CU/MCI/DEM)	58/54/8	11/38/15	1/7/15	1/10/5	1/14/3	<0.001^1,2,3,4^
APOE4 carrier^a^ (−/+)	72/22	23/19	4/9	5/4	4/8	<0.001^2,4^
Neuropsychological tests^b^						
MMSE, total score	26.8 (3.37)	23.9 (5.57)	21.6 (6.08)	24.9 (2.85)	26.1 (2.13)	<0.001^1,2,3,5,9^
FCSRT immediate recall [NA]	14.8 (1.88) [28]	13.6 (2.49) [21]	11.3 (4.53) [14]	12.4 (3.91) [7]	12.2 (3.63) [5]	<0.001^1,2,3,4^
FCSRT delayed free recall [NA]	9.39 (4.12) [32]	5.71 (4.28) [33]	6.80 (5.22) [18]	6.88 (4.19) [8]	3.50 (3.51) [10]	<0.001^1,4^
FCSRT delayed total recall [NA]	14.7 (2.09) [32]	11.6 (4.56) [33]	12.4 (3.36) [18]	12.1 (3.94) [8]	10.2 (3.11) [10]	<0.001^1,3,4^
TMT-A [NA]	48.2 (23.1) [34]	57.0 (34.8) [24]	66.4 (22.6) [13]	73.8 (28.6) [7]	57.5 (21.7) [7]	0.022^2,3,6^
TMT-B [NA]	111 (55.3) [38]	145 (69.0) [31]	212 (117) [19]	189 (97.0) [7]	147 (75.6) [9]	<0.001^1,2,3^
Semantic fluency [NA]	16.5 (5.72) [52]	15.1 (5.26) [31]	8.90 (5.11) [13]	12.8 (5.17) [4]	15.4 (4.70) [7]	0.001^2,3,5,9^
Phonemic fluency [NA]	15.9 (6.11) [53]	14.9 (6.05) [32]	13.5 (4.78) [15]	15.2 (7.26) [4]	15.6 (6.64) [7]	0.842
Digit span [NA]	46.4 (16.0) [33]	41.3 (15.1) [24]	29.7 (13.0) [11]	34.2 (10.2) [7]	39.8 (11.3) [9]	0.002^2,3,5^
Neuroimaging biomarkers						
Hippocampal volume	3,831 (456)	3,361 (527)	3,477 (470)	3,324 (274)	3,352 (573)	<0.001^1,2,3,4^
Amyloid status^a^ (−/+)	83/22	12/49	1/19	1/9	0/16	<0.001^1,2,3,4^
Amyloid centiloid^a^	9.90 (29.2)	67.2 (44.5)	89.2 (24.9)	91.3 (30.8)	81.4 (32.2)	<0.001^1,2,3,4^
Tau status (−/+)	120/0	25/39	0/23	0/16	0/18	<0.001^1,2,3,4,5,6,7^
Global tau SUVR	1.14 (0.11)	1.47 (0.28)	1.83 (0.34)	1.53 (0.33)	1.51 (0.22)	<0.001^1,2,3,4,5,8,9^

Abbreviations: CU = cognitively unimpaired; DEM = dementia; FCSRT = Free and Cued Selective Reminding Test; MCI = mild cognitive impairment; MMSE = Mini-Mental State Examination; MTL = medial temporal lobe; NA = not available data; SUVR = standardized uptake value ratio; TMT = Trail-Making Test.

Significant differences at posthoc comparisons (*p* < 0.05): ^1^S0 vs S1, ^2^S0 vs S2, ^3^S0 vs S3, ^4^S0 vs S4, ^5^S1 vs S2, ^6^S1 vs S3, ^7^S1 vs S4, ^8^S2 vs S3, ^9^S2 vs S4, ^10^S3 vs S4.

a Amyloid data were available in a subset of 212 participants; APOE data were available in a subset of 170 participants.

b Neuropsychological tests were available in a subset of participants with sample size depending on each test, and the number of missing values is reported in the brackets.

Participants had an average baseline MMSE of 24.67 ± 3.02. One hundred thirty-five participants had a follow-up after an average interval of 26.68 ± 12.82 months.

### Visual Subtypes and Inter-Reader Agreement

All scans were visually classified into subtypes, except for 4 cases because of non-AD patterns, namely scans with cortical uptake above background in other regions not considered by Braak.^21^ In the resulting sample of 241 participants (133 with a clinical follow-up), 120 scans were visually read as negative (S0, 50%), 64 scans were visually classified as limbic (S1, 27%), 23 as MTL-sparing (S2, 10%), 16 as posterior (S3, 6%), and 18 as lateral temporal (S4, 7%) subtypes. Descriptive statistics for each visual subtype are presented in Table 1 and the comparison between the subtypes. In patients with amyloid-positive MCI and DEM, we did not find any significant association between visual SuStaIn subtypes and typical/atypical clinical AD variants (χ^2^ = 3.02, *p* > 0.05).

**Figure 2:**
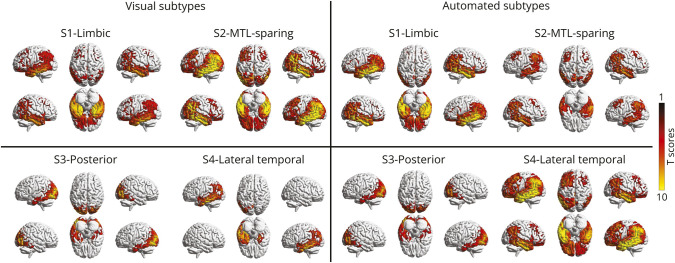
Tau Accumulation Patterns at Group Level for Each Visual and Automated Subtype Thresholded SPM images (*p* = 0.05 family-wise error-corrected at the cluster level, minimum cluster extent κ = 100 voxels) are overimposed on a standard ICBM152 surface with BrainNet Viewer. S1 corresponds to the limbic subtype, S2 to medial temporal lobe-sparing subtype, S3 to posterior subtype, and S4 to lateral temporal subtype. For visual S4, only left-lateralized tau patterns were considered for visualization purposes. MTL = medial temporal lobe.

Tau patterns at group level for each visual subtype confirmed the presence of typical AD pattern in S1, hippocampal-sparing pattern with widespread cortical tau accumulation in S2, posterior occipital pattern in S3, and lateral temporal pattern in S4 (Figure 2).

Our analysis revealed good inter-rater reliability in the visual classification of subtypes (κ = 0.65, *p* < 0.001), and changes between the 2 raters' classification are fully reported in eFigure 3. A good inter-rater agreement was found in the visual rating of each region (eTable 1). According to the visual consensus classification, individuals across all 4 subtypes showed a higher frequency of MCI and DEM diagnoses and amyloid- and tau-positivity compared with negative (S0) individuals (Table 1). Individuals across all 4 subtypes, except for lateral temporal subtype (S4), expressed worse baseline global cognition than negative (S0) (Table 1). Individuals with MTL-sparing subtype (S2) were younger than limbic (S1) and posterior (S3) subtypes' individuals, had worse MMSE scores and phonemic fluency than negative (S0), limbic (S1), and lateral temporal (S4), and showed higher global tau than all other subtypes (Table 1).

**Figure 3:**
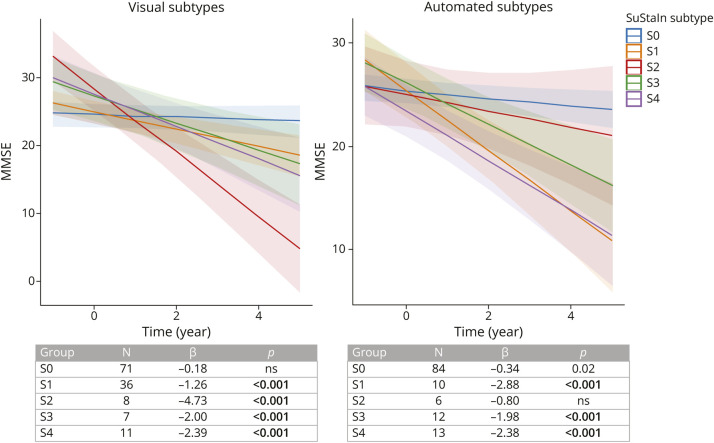
Longitudinal Results The plots show different cognitive trajectories of MMSE scores over time in the different subtypes, defined visually (left) and algorithm-based (right). Age, sex, clinical stages, and visual tau Braak stages were included as covariates in the analysis. S1 corresponds to the limbic subtype, S2 to medial temporal lobe-sparing subtype, S3 to posterior subtype, and S4 to lateral temporal subtype. MMSE = Mini-Mental State Examination.

Longitudinal findings obtained with linear mixed-effect models correcting for age, sex, clinical stages, and Braak visual stages showed that individuals across all 4 positive subtypes displayed a significantly steeper cognitive decline over time compared with negative individuals (S0) (*p* < 0.001) (Figure 3). MTL-sparing subtype (S2) showed the steepest cognitive decline over time, significantly different compared with other groups' (*p* < 0.001) when we considered limbic subtype (S1) as the reference group in the model. The results were consistent with and without including Braak visual stages or SuStaIn stages and education as covariates in the longitudinal models (data not shown).

### Automated Subtypes and Intermethod Agreement

Out of the total sample, 211 patients were classified into automated subtypes using SuStaIn algorithm. We could not classify all participants using the algorithm because of missing MRI images for coregistration and failures in preprocessing (segmentation) steps. According to the automated classification, 132 scans were negative (S0, 62%), 19 scans were classified as limbic (S1, 9%), 21 as MTL-sparing (S2, 11%), 14 as posterior (S3, 6%), and 18 as lateral temporal (S4, 12%). Descriptive statistics and differences between automated subtypes are displayed in Table 2. In patients with amyloid-positive MCI and DEM, we did not find any significant association between automated subtypes and typical/atypical clinical AD variants (χ^2^ = 4.84, *p* > 0.05).

**Table 2:** Clinical Features of Automated Subtypes

	S0: Negative (N = 132)	S1: Limbic subtype (N = 19)	S2: MTL-sparing subtype (N = 21)	S3: Posterior subtype (N = 14)	S4: Lateral temporal subtype (N = 24)	*p* Values
Demographic features						
Age, y	70.2 (8.68)	74.4 (5.85)	67.6 (13.2)	72.0 (4.64)	68.7 (10.8)	0.139
Sex (women/men)	60/72	11/8	12/9	10/4	14/10	0.272
Education, y	14.0 (4.36)	13.1 (3.87)	13.2 (3.48)	13.9 (3.52)	13.0 (4.53)	0.768
Diagnostic stages (CU/MCI/DEM)	56/65/11	2/11/5	5/11/5	1/9/4	0/16/8	<0.001^1,2,3,4^
APOE4 carrier^a^ (−/+)	80/26	3/6	5/10	4/8	2/9	<0.001^2,3,4^
Neuropsychological tests^b^						
MMSE, total scores	26.7 (3.01)	24.4 (3.53)	24.4 (4.57)	24.9 (2.96)	21.3 (7.71)	<0.001^1,4^
FCSRT immediate recall [NA]	14.6 (2.11) [27]	12.0 (3.98) [5]	12.5 (3.67) [10]	13.0 (3.54) [2]	12.9 (2.47) [12]	0.001^1,2,3,4^
FCSRT delayed free recall [NA]	8.61 (4.33) [33]	4.43 (3.82) [12]	5.67 (4.41) [15]	5.18 (2.56) [3]	6.83 (5.74) [18]	0.010^1,3^
FCSRT delayed total recall [NA]	14.0 (2.90) [33]	10.4 (3.78) [12]	11.8 (6.11) [15]	11.6 (2.50) [3]	11.5 (4.32) [18]	0.003^1,3^
TMT-A [NA]	49.9 (24.8) [32]	58.1 (23.6) [7]	75.0 (49.8) [10]	59.2 (18.6) [1]	49.7 (20.4) [14]	0.043^2,3^
TMT-B [NA]	117 (60.4) [39]	122 (55.9) [11]	156 (74.3) [13]	145 (65.0) [3]	164 (98.7) [16]	0.131
Semantic fluency [NA]	16.5 (5.65) [59]	15.9 (7.29) [10]	13.5 (6.40) [11]	12.3 (4.42) [4]	11.8 (4.78) [12]	0.023^3,4^
Phonemic fluency [NA]	15.4 (6.63) [61]	16.0 (5.79) [10]	15.4 (5.39) [12]	16.2 (6.18) [5]	15.2 (6.69) [12]	0.994
Digit span [NA]	45.5 (15.6) [33]	44.9 (12.2) [9]	40.6 (14.4) [12]	36.8 (12.2) [1]	38.2 (14.6) [12]	0.192
Neuroimaging biomarkers						
Hippocampal volume	3,759 (490)	3,138 (614)	3,425 (534)	3,397 (523)	3,421 (378)	<0.001^1,2,3,4^
Amyloid status^a^ (−/+)	85/37	1/16	1/18	2/11	0/22	<0.001^1,2,3,4^
Amyloid centiloid^a^	18.7 (36.9)	75.0 (41.5)	82.9 (31.9)	86.6 (41.8)	93.7 (23.3)	<0.001^1,2,3,4^
Tau status (−/+)	117/13	2/17	3/18	2/12	0/23	<0.001^1,2,3,4^
Global tau SUVR	1.16 (0.13)	1.72 (0.19)	1.50 (0.22)	1.69 (0.36)	1.69 (0.29)	<0.001^1,2,3,4^

Abbreviations: CU = cognitively unimpaired; DEM = dementia; FCSRT = Free and Cued Selective Reminding Test; MCI = mild cognitive impairment; MMSE = Mini-Mental State Examination; MTL = medial temporal lobe; NA = not available data; SUVR = standardized uptake value ratio; TMT = Trail-Making Test.

Significant differences at posthoc comparisons (*p* < 0.05): ^1^S0 vs S1, ^2^S0 vs S2, ^3^S0 vs S3, ^4^S0 vs S4, ^5^S1 vs S2, ^6^S1 vs S3, ^7^S1 vs S4, ^8^S2 vs S3, ^9^S2 vs S4, ^10^S3 vs S4.

a Amyloid data were available in a subset of 193 participants; APOE data were available in a subset of 153 participants.

b Neuropsychological tests were available in a subset of participants with sample size depending on each test, and the number of missing values is reported in the brackets.

All analyses including the visual classification were also run in this subsample (n = 211), obtaining comparable results (eTable 2 and eFigure 4).

**Figure 4:**
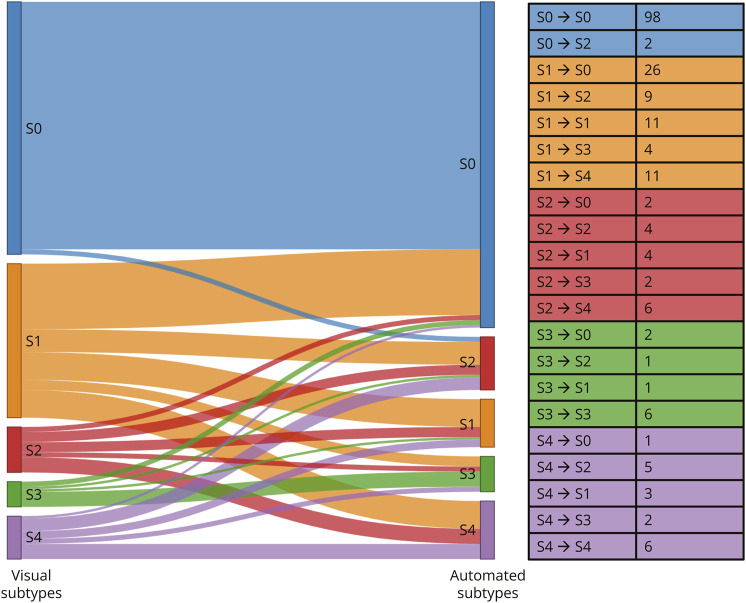
Visual and Automated Classifications Sankey plot depicts the subtypes' changes from visual to automated classifications, wherein the line width indicates the number of participants that is fully reported in the table. S1 corresponds to the limbic subtype usually fitting the Braak staging system, S2 to medial temporal lobe-sparing subtype, S3 to posterior subtype, and S4 to lateral temporal subtype.

Considering the whole sample, the automated classification showed a fair agreement with visual classification (κ = 0.39, *p* < 0.001), with automated classification assigning more negative (S0, 62.86%) than visual classification (48.54%) (Figure 4). Most cases in Braak stages I–III, according to the visual rating, were classified negative (S0) by the automated classification (81%), whereas the visual rating classified them as limbic (S1, 92%). When we excluded subjects in Braak stages I–III to focus on advanced stages, a higher agreement of κ = 0.48 (*p* < 0.001) between automated and visual subtypes was reached. When we tested the 2 methods agreement within different clinical stages, we found a fair agreement for CU (κ = 0.39, *p* < 0.001) and MCI (κ = 0.38, *p* < 0.001) and a lower one for DEM (κ = 0.18, *p* < 0.001) that represents also the smallest group. When we focus on clinical AD atypical variants, only 12% were consistently classified by the 2 methods. Taking into consideration only cases that have been classified by the algorithm with a probability higher than 50% (n = 73), the agreement did not increase (κ = 0.26, *p* < 0.001); whereas when we considered only cases that have been classified with high confidence by both raters (n = 159), the agreement between the 2 methods reached a κ of 0.489 (*p* < 0.001).

Tau patterns at group level for each automated subtype are similar to the visual subtypes' pattern (Figure 2). However, visual read tends to be less likely to assign high tau people to limbic subtype (S1) and seems to require quite marked asymmetry to capture lateral temporal (S4) than the SuStaIn algorithm.

A good correlation was found between global tau SUVR and progressive stages (1–30) that are probabilistically assigned by SuStaIn algorithm to individuals along the 4 subtype trajectories (*r* = 0.887, *p* = 0.001), with these stages progressively increasing with the increasing of visual Braak stages.

According to the automated classification, individuals across all 4 subtypes showed a higher frequency of MCI and DEM diagnoses and amyloid-positivity and tau-positivity compared with negative (S0) individuals (Table 2). Only individuals with limbic (S1) and lateral temporal (S4) subtypes expressed significantly worse global cognition than S0 (Table 2).

The longitudinal results showed that individuals with limbic (S1), posterior (S3), and lateral temporal (S4) displayed significantly steeper cognitive decline over time compared with negative (S0) (*p* < 0.001) (Figure 3). Limbic subtype (S1) showed significantly steeper cognitive decline over time than negative (S0) and MTL-sparing (S2) subtype (*p* < 0.001) when we considered limbic (S1) as reference group. The results were consistent with and without including Braak visual stages and education as covariates (together with age, sex, and clinical stages) in the longitudinal models (data not shown).

### Discordant and Concordant Cases

Eighty-five of 210 cases (40%) were classified into different visual and automated subtypes (discordant cases), while 125 cases (60%) received an identical classification by the 2 approaches (concordant cases). Discordant cases included individuals with significantly higher frequency of MCI and DEM diagnosis, worse global cognition scores, and higher amyloid and tau loads than concordant cases (eTable 3). 30% of discordant cases (26/85) were visually classified as limbic (S1) but classified as negative by the automated SuStaIn that seems to be more conservative. 13% of discordant cases (11/85) were visual limbic subtype (S1) and automated lateral temporal subtype (S4). 10% of discordant cases (9/85) were visual limbic subtype (S1) and automated MTL-sparing subtype (S2). The other changes represented lower percentages (Figure 4). Regarding the proportion of agreement within subtypes, the only concordant subtype was the posterior (S3).

When we looked at whether the raters' confidence and/or the algorithm probability could explain the difference between discordant and concordant cases, we found that (1) 47 discordant cases have been classified by the algorithm with a probability higher than 50%, (2) the algorithm probability did not differ significantly for discordant and concordant cases (*p* = 0.53), (3) the agreement between the 2 raters did not differ (*p* = 0.08), and (4) the confidence of a single rater was slightly higher for concordant cases compared with discordant ones (*p* = 0.04). These analyses have been run excluding negative individuals (S0) for whom the algorithm probability is not calculated.

## Discussion

Although the progression of tau pathology in AD has long been described by the spatiotemporal spreading from the entorhinal cortex to the neocortex,^25^ substantial variability in tau accumulation has now become accepted given the relatively high frequency of cases that do not fit Braak staging.^9^ Recently, SuStaIn showed that cortical spread of tau pathology is better described by a data-driven model including multiple spatiotemporal trajectories.^14^ Based on SuStaIn, we developed and tested a novel topographic visual method for tau-PET (Figure 1), reaffirming the existence of multiple tau accumulation patterns in a clinical setting and setting a standard for subtypes' clinical translation. The visually classified tau SuStaIn subtypes differed in global tau loads, clinical characteristics, and long-term outcomes. The visual classification was able to detect individuals with a faster decline, supporting its clinical usefulness for the detection of higher-risk AD patterns. Specifically, we found the hippocampal-sparing pattern in S2 subtype had the worst cognitive and biomarkers profile and the most rapid short-term cognitive progression.

A substantial agreement between raters was found in visually interpreting tau subtypes (κ > 0.65, *p* < 0.001), supporting a good reproducibility of the visual method in clinical practice. Our visual method classified 50% of cases as negative, 27% as limbic (S1), 10% as MTL-sparing (S2), 6% as posterior (S3), and 7% as lateral temporal (S4). A similar frequency of subtypes resulted from the automated classification and a fair agreement between visual and automated classifications was observed (κ = 0.39, *p* < 0.001). However, the automated approach classified negative scans at a higher rate, and most discordances and lower inter-methods agreement occurred for individuals with advanced disease stages, already diagnosed with DEM, for whom the visual rating seemed also to be less confident. Instead, the algorithm probability was similar between concordant and discordant cases. We noticed that the algorithm was not sensitive to uptake limited to MTL, and detected more frequently an asymmetrical uptake, thus leading to a higher number of participants classified as lateral temporal (S4). The large source of discrepancy because of limbic-negative (S1–S0) could be partially explained by a rather conservative threshold for positivity for the algorithm, that is also regionally unbiased without a hyperfocus on the MTL over other regions. An automated algorithm likely errs on the side of conservatism, but being able to read early-stage tau scans with more confidence represents an advantage of human readers. Instead, for lateral temporal subtype (S4), the human threshold for asymmetry is likely less subtle, suggesting a bias in the visual read. Moreover, in cases with more advanced pathology, the ability to visually distinguish different subtypes might be lower than semiquantitative indices given that all subtypes converge in widespread neocortical tau pattern at later disease stages.^14^

In line with the previous SuStaIn article,^14^ the limbic subtype (S1) with MTL tau pathology and Braak-like progression^25^ were the most frequent and presented with typical AD features, including amnestic profile, impairment in global cognition, hippocampal atrophy, and amyloid positivity. The limbic subtype showed steeper cognitive decline than tau negative individuals, according to both visual and automated classifications, but presented a less severe cognitive decline than the MTL-sparing subtype, only using the visual classification (Figure 3). This is in line with the fact that greater neocortical tau burden and less hippocampal tau in histopathologic^9,26^ and PET studies^12,27-29^ have been associated with atypical AD cases that exhibit early-onset cognitive and functional impairment and a more rapid clinical decline.^30^

According to our visual classification, the MTL-sparing subtype (S2) represents the most malignant subtype, including younger individuals with lower global cognition and verbal fluency scores and higher global tau load, but without amyloid load nor hippocampal atrophy, compared with other subtypes. This clinical profile was consistent with previous publications.^11,14,31^ The concept of a hippocampal-sparing subtype of AD challenges the widely accepted model of tau spreading from the entorhinal to the associative cortices^25^ in favor of a less common pathway starting from multimodal association cortices with an eventually late limbic involvement.^32^ The hippocampal-sparing subtype has usually been defined postmortem as relatively higher neurofibrillary tau tangle counts in the association cortex and lower hippocampal counts^9^ and in vivo cases with MTLs spared relative to the high cortical tracer retention visualized at PET scans.^31^ On the other hand, neuropathologic studies did not find any individual case with tau in the association cortex while completely sparing the hippocampus.^9,10,33,34^ Importantly, when comparing imaging and neuropathologic data, it should be emphasized that the ability of PET to identify accumulation in MTL is less validated than in the neocortex and might be impaired by adjacent off-target binding in the choroid plexi and low spatial resolution.^4^ We did find MTL-sparing (S2) individuals with MTL tau accumulation, but it was less severe than in the association cortex and limbic subtype (S1) (Figure 2). Our results also argued in favor of worse prognosis of the hippocampal-sparing subtype, with steeper cognitive decline than other subtypes (Figure 3). This result fits well with the hypothesis of a greater effect of neurofibrillary pathology in the neocortex than hippocampal pathology on global cognitive decline, as suggested by previous studies.^9,11,33,35^ Moreover, neurofibrillary tau tangle density in association cortices relative to hippocampal structures have been found to be greater in postmortem brains of early-onset patients,^36^ consistent with the observation of younger participants in MTL-sparing subtype in our sample.

According to both visual and automated classifications, posterior (S3) and lateral temporal (S4) subtypes represented a small proportion of our memory clinic sample: 6% and 7%–12%, respectively. Posterior subtype (S3) presented with significant tau pathology in posterior brain regions (including occipital), but also less MTL and frontal binding, while lateral temporal subtype (S4) showed both temporoparietal and MTL tau with marked lateralization (Figure 2). Common variation in occipital tau pathology has been described in the literature in both preclinical and symptomatic AD. For example, hyperphosphorylated tau-immunoreactive AD-related tau pathology has been reported in visual association cortex even in cognitively intact participants either lacking pathology elsewhere or displaying pathology restricted only to the entorhinal region.^37^ However, the clinical meaning of these observations, and their possible relationship with PCA, remains unclear. Given the close relationship between tau topographical distribution and clinical manifestations of AD,^12,38,39^ it is reasonable that PCA and lvPPA resemble the clinical extreme of posterior (S3) and lateral temporal (S4) tau subtypes.^14,40^ However, we could not find any significant association between the frequency of AD atypical variants and subtype assignment, likely because most of our patients with AD had a typical clinical profile. Interestingly, we found only a few cases (12%) consistently classified by the automated and visual classification when focusing only on AD clinical variants.

The existence of tau accumulation subtypes confirmed here by visual assessment in a clinical setting supports selective vulnerabilities to tau pathology that need further investigation to elucidate their clinical and pathophysiologic significance. The most compelling hypothesis to explain the substantial heterogeneity in tau patterns implicates the brainSupplementss network architecture at system-level pathophysiology of neurodegenerative diseases.^41^ According to that hypothesis, pathologic proteins accumulate along specific macroscale brain networks and tau accumulates preferentially in regions closely connected to the individualized epicenters in AD.

This study has several limitations. First, we proposed a visual clinical applicable method inspired by the 4 subtypes previously identified by SuStaIn, rather than a robust clinical replication of the latter. For this reason, we did not take into consideration some aspects, namely 30 SuStaIn stages, given their limited clinical applicability, to develop our flowchart. Second, the partial clinical and neuropsychological characterization of patients limits a more detailed evaluation of clinical profiles and the relatively small number of clinical follow-ups available per subtypes limits strong prognostic implication. Third, the visual method proposed here should be replicated using second-generation tau-PET ligands (^18^F-MK6240, ^18^F-PI2620, and ^18^F-RO948) presenting higher sensitivity to tau and less off-target binding. Finally, although our sample includes many individuals from memory clinic setting that improves clinical applicability of our results, the visual method should be confirmed in other large cohorts.

In conclusion, the visual method proposed here was able to identify different tau accumulation patterns, could be easily implemented clinically, and could benefit clinical practice in identifying high-risk AD patterns and clinical trials in monitoring different tau accumulation subtypes.

## Glossary

AD: Alzheimer disease
CU: cognitively unimpaired
DEM: dementia
FDA: US Food and Drug Administration
lvPPA: logopenic variant primary progressive aphasia
MCI: mild cognitive impairment
MMSE: Mini-Mental State Examination
MTL: medial temporal lobe
PCA: posterior cortical atrophy
ROI: region of interest
SuStaIn: Subtype and Stage Inference
SUVR: standardized uptake value ratio
